# Organic-Inorganic Co-Modified PVDF Membrane for High-Flux Oil/Water Separation and Simultaneous Multi-Pollutant Removal

**DOI:** 10.3390/molecules31081372

**Published:** 2026-04-21

**Authors:** Jie Teng, Zekai Lu, Xiangbo Ma, Wencheng Zhu, Yongqiang Yang, Pu Li, Xia Xu

**Affiliations:** 1 School of Urban Construction, Changzhou University, Changzhou 213164, China; tammytengjie@cczu.edu.cn (J.T.); luzekai0926@163.com (Z.L.); mmmaxiangbo@163.com (X.M.); 18921526303@163.com (W.Z.); lipuhku@cczu.edu.cn (P.L.); 2 Poseidon (JiangSu) New Material Technology Co., Ltd., Changzhou 213002, China; dyqyang6@163.com

**Keywords:** PVDF membrane, oil-water separation, heavy metal removal, superhydrophilicity

## Abstract

The coexistence of emulsified oil, dissolved organics, and heavy metal ions in industrial oily wastewater makes one-step treatment highly challenging. Herein, an organic-inorganic co-modified PVDF composite membrane (MTSP) was fabricated via nonsolvent-induced phase separation, with tea polyphenols, SiO_2_, and fibrous MXene synergistically incorporated. The resulting membrane exhibited a superhydrophilic/underwater oleophobic surface, with a water contact angle of 1° and an underwater oil contact angle of ~136°, owing to the optimized surface chemistry and hierarchical pore structure. As a result, the MTSP membrane effectively suppressed oil fouling while enabling rapid water transport. At 0.1 bar, the optimized membrane delivered an oil/water separation efficiency of ~99.5% and a high flux of 2420–2670 L·m^−2^·h^−1^, while maintaining >99% separation efficiency for various emulsified oils, including kerosene, edible oil, n-hexane, and 1,2-dichloroethane. It also showed excellent recyclability and chemical stability, retaining >98–99% efficiency after five cycles and after 24 h exposure to pH 1 and pH 12 conditions. Notably, for complex simulated wastewater containing emulsified kerosene, phenol, and Fe^3+^, Cu^2+^, Zn^2+^, and Cd^2+^, the membrane maintained ~99% oil/water separation efficiency and simultaneously removed ~79% of phenol and 70–86% of heavy metal ions in a single filtration process. The superior performance is attributed to the synergistic effects of the superhydrophilic/underwater-oleophobic membrane surface, hierarchical transport channels enabling rapid water permeation, and multifunctional sites that adsorb/coordinate dissolved pollutants. This work provides a simple, scalable design strategy for PVDF-based membranes that integrate high-flux separation, antifouling performance, and multi-pollutant remediation for the treatment of complex oily wastewater.

## 1. Introduction

The rapid growth of petroleum extraction, mechanical processing, food manufacturing, and chemical production has led to a continuous increase in oily wastewater generation [1]. In real industrial scenarios, such effluents rarely consist solely of dispersed particles but commonly contain surfactant-stabilized oil-in-water emulsions, dissolved organic contaminants, and metal ions [2,3,4,5]. The compositional complexity and physicochemical stability of these multicomponent systems make their treatment particularly challenging [5,6,7]. Among the available treatment technologies, membrane separation has emerged as one of the most promising approaches for oily wastewater treatment because of its operational simplicity, relatively low energy consumption, high separation efficiency, and compatibility with modular process integration [8,9,10].

Poly(vinylidene fluoride) (PVDF) is a widely used membrane matrix material for oil/water separation. Its popularity arises from excellent chemical resistance, thermal stability, mechanical robustness, and strong film-forming properties [11,12]. PVDF is particularly compatible with mature fabrication methods. For instance, nonsolvent-induced phase separation supports scalable membrane production [13]. However, pristine PVDF is intrinsically hydrophobic due to its low surface energy [14]. This hydrophobicity helps maintain material stability while also promoting the adsorption of oil droplets and hydrophobic organic species during separation. As a result, severe membrane fouling, pore blockage, and rapid flux decline occur [15,16,17]. Furthermore, unmodified PVDF membranes primarily rely on size exclusion. Thus, their capacity for active removal of dissolved organic pollutants and metal ions remains limited [18]. These intrinsic drawbacks limit their use for the one-step treatment of complex industrial oily wastewater [19]. Therefore, there is considerable scientific and practical interest in enhancing the hydrophilicity, antifouling performance, and multifunctional contaminant-removal capabilities of PVDF membranes. It is also important to preserve processability and structural stability [20].

To address these challenges, various modification strategies have been developed for PVDF membranes. These include surface grafting, functional coating, nanofiller incorporation, and bioinspired interfacial engineering [21,22,23]. These strategies can be implemented through different routes, such as surface deposition/coating, in situ growth, layer-by-layer assembly, or direct blending into the casting solution during nonsolvent-induced phase separation (NIPS). Among these, polyphenolic compounds have attracted attention due to their natural abundance, high phenolic hydroxyl content, and strong interfacial adhesion properties [24]. Tea polyphenols (TP) can improve membrane wettability and provide abundant functional sites. This occurs through hydrogen bonding, coordination, and π-π interactions, enhancing fouling resistance and adsorption capacity [25,26,27]. Inorganic nanomaterials also improve the surface hydrophilicity, roughness, and structural integrity of PVDF membranes [28]. SiO_2_ stands out because of its high density of surface hydroxyl groups, chemical stability, and tunable particle size. These features help strengthen interfacial hydration and tailor nanostructured surfaces, thereby improving wetting and permeation performance [29]. MXenes, a family of two-dimensional transition-metal carbides/nitrides, exhibit many surface terminal groups, a high specific surface area, and excellent hydrophilicity. This makes them promising for membrane-based separation and water treatment [30,31,32]. Incorporating MXene provides functional transport interfaces for water molecules and enhances interfacial repulsion against oil droplets and other pollutants [33].

Motivated by these considerations, the construction of an organic-inorganic co-modified PVDF membrane through a one-step blending/NIPS route represents an effective and practical strategy [34,35,36]. In this route, TP, SiO_2_, and fibrous MXene are co-incorporated into the PVDF casting solution before phase inversion, so that membrane formation and functionalization can be achieved simultaneously in a single process. This also allows the functional additives to be introduced during membrane formation rather than in a separate post-treatment step [37,38]. Compared with post-coating or sequential surface modification, such an incorporation process is more favorable for integrating functional components with the membrane matrix, while also enabling concurrent regulation of interfacial properties and pore structure during membrane formation. This route also avoids additional modification steps after membrane fabrication and is therefore more compatible with simple and scalable PVDF membrane production [39,40]. As a result, the obtained membrane is expected to develop a stable hydration interface, hierarchical transport pathways for rapid water permeation, and multifunctional sites for pollutant capture, thereby achieving oil repellence, high permeation flux, improved antifouling behavior, and the simultaneous removal of multiple classes of contaminants [41,42,43,44,45].

Based on this rationale, this study employed a one-step non-solvent induced phase separation (NIPS) strategy to fabricate an organic-inorganic hybrid PVDF membrane(MTSP) through the synergistic incorporation of TPs, SiO_2_, and fibrous MXene. The membrane was systematically characterized in terms of its microstructure, surface chemistry, wettability, oil/water separation performance, cyclic stability, and acid/base resistance. In addition, its capacity to simultaneously removal phenolic compounds and multiple heavy metal ions from complex simulated wastewater was evaluated. This work is expected to provide a useful design framework for PVDF-based functional membranes that can integrate high flux, fouling resistance, and multipollutant remediation in oily wastewater treatment.

## 2. Results and Discussion

### 2.1. Membrane Characterization

**Figure 1:**
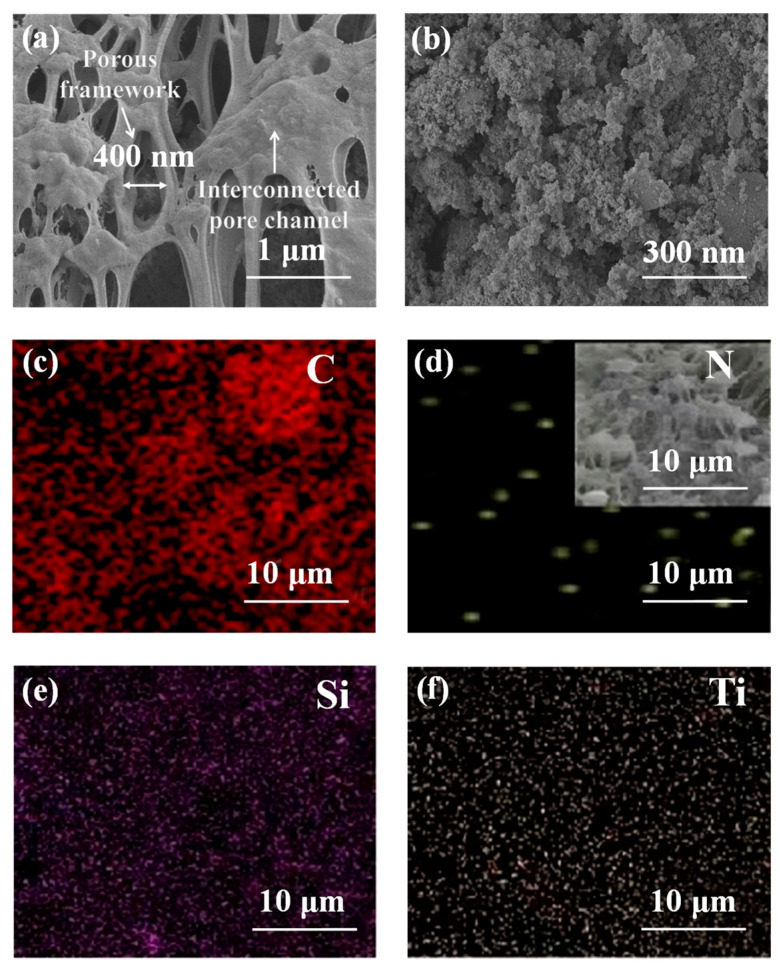
SEM images of MTSP membrane at (**a**) 10,000× and (**b**) 50,000× magnifications. (**c**–**f**) EDS mapping images of C, N, Si, and Ti elements at 2000× magnification.

SEM images were used to examine the morphology of the MTSP membrane. As shown in Figure 1a, the cross-section exhibits a typical asymmetric porous structure formed by the NIPS process, characterized by interconnected macrovoids and microporous substructures. The macrovoid channels are on the micrometer scale and remain continuous throughout the cross-section, indicating a well-preserved porous framework. At higher magnification (Figure 1b), the membrane surface displays a rough and textured morphology composed of aggregated nanoscale features rather than a smooth surface. These observations indicate that the incorporation of TP-SiO_2_-fibrous MXene modifies the surface morphology while preserving the interconnected porous structure of the membrane, which is consistent with the enhanced wettability and separation performance discussed below.

**Figure 2:**
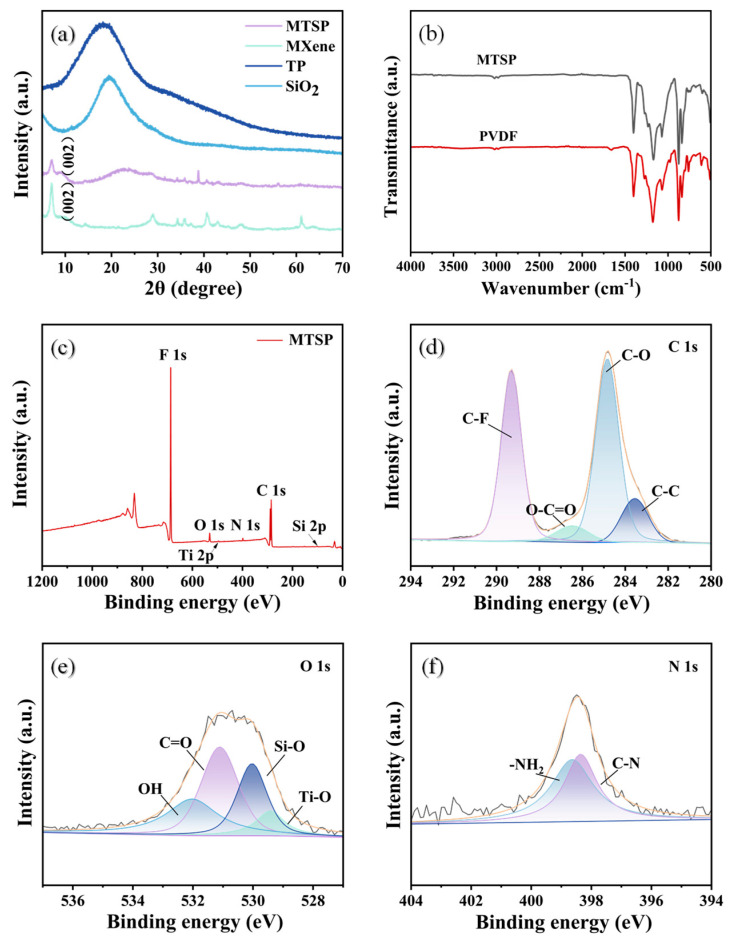
(**a**) XRD and (**b**) FTIR of MTSP. (**c**) XPS survey spectrum of MTSP. (**d**) The C1s peak, (**e**) the O1s peak, and (**f**) the Si 2p peak of XPS.

XRD analysis (Figure 2a) confirms the successful incorporation of the components. The characteristic (002) peak of MXene appears at 2θ ≈ 9.2°, indicating that its layered structure is retained after blending. In the composite membrane, this peak shows slight broadening and a small shift, suggesting reduced stacking coherence and partial delamination of MXene layers, rather than the formation of a new crystalline phase. Given that the (002) reflection of Ti_3_C_2_T_x_ is sensitive to interlayer spacing and stacking order, these changes are consistent with weakened layer stacking and enhanced interfacial interaction with the polymer matrix [46]. In contrast, TP and SiO_2_, as amorphous components, contribute to broad humps in the range of 15–30° without generating additional crystalline peaks, indicating their amorphous nature and uniform dispersion within the membrane during phase inversion.

The FTIR spectra (Figure 2b) further support the incorporation of the functional components into the MTSP membrane. The pristine PVDF membrane exhibits characteristic C–F stretching bands at 1179 and 1071 cm^−1^, which remain visible in the MTSP membrane, indicating that the main polymer backbone is preserved after modification. In addition, the absorption bands in the 600–800 cm^−1^ region (e.g., 764 and 874 cm^−1^) are associated with the characteristic vibrational modes of crystalline PVDF, suggesting that the PVDF matrix retains its crystalline features. Compared with pristine PVDF, the MTSP membrane shows additional spectral features attributable to the introduced functional components. In particular, a weak broad band associated with –OH stretching vibrations from TP and oxygen-containing groups on fibrous MXene can be identified more clearly in the enlarged 3000–3700 cm^−1^ region (Figure S1a). Likewise, Si–O–Si/Si–OH related features can be more readily distinguished in the enlarged 900–1200 cm^−1^ window (Figure S1b), despite partial overlap with the intrinsic PVDF bands [47]. A band in the 500–600 cm^−1^ region may also be related to Ti-based vibrations from fibrous MXene. Taken together, these spectral changes, along with the preserved characteristic bands of PVDF, indicate the successful incorporation of TP, SiO_2_, and fibrous MXene into the membrane.

Elemental composition analysis by XPS and EDS supports the successful embedding of all components (Figure 1c–f and Figure 2c–f). The XPS survey spectrum reveals the presence of F, C, O, N, Si, and Ti, corresponding to the PVDF matrix and the functional additives. High-resolution XPS spectra further identify surface oxygenated and nitrogenous groups (C–O, C=O, –OH, –NH), consistent with polyphenol grafting and MXene modification. EDS mapping shows a homogeneous distribution of these elements across the membrane surface and internal pore walls. Collectively, these results confirm that TP, SiO_2_, and MXene were effectively incorporated into the membrane matrix, forming a structurally robust, chemically functionalized composite membrane that provides a foundation for enhanced wettability and separation performance.

### 2.2. Surface Wettability and Anti-Fouling Behavior

**Figure 3:**
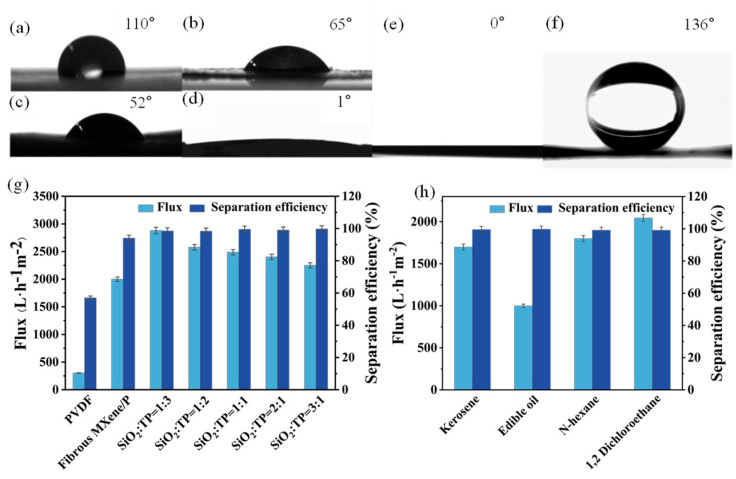
WCAs on the surfaces of (**a**) PVDF, (**b**) PVDF@SiO_2_, (**c**) PVDF@TP, (**d**) MTSP. UOCA of (**e**) PVDF and (**f**) MTSP. Oil-water separation efficiency and flux of (**g**) various membranes and (**h**) MTSP membrane.

Surface wettability is a crucial factor governing membrane separation performance for oil/water systems. The pristine PVDF membrane is hydrophobic, exhibiting a water contact angle (WCA) of about 110° (Figure 3a). Each sequential modification markedly improves hydrophilicity. After incorporating hydrophilic SiO_2_ nanoparticles, the WCA drops to ~65° (Figure 3b), and with TP coating, it further decreases to ~52° (Figure 3c). Strikingly, the fully loaded MTSP composite membrane becomes superhydrophilic, with a WCA approaching 1° (Figure 3d). This transition from hydrophobic to superhydrophilic behavior is mainly attributed to the abundant hydrophilic groups introduced by TP and fibrous MXene, which promote rapid water spreading on the membrane surface. The enhanced hydrophilicity enables the MTSP membrane to form a stable hydration layer when immersed in water, which is critical for its anti-oil-fouling behavior. In addition, previous studies on related fibrous MXene/SiO_2_-modified PVDF systems have shown that such hybrid surface modification can also induce increased surface roughness, which may further amplify the intrinsic wettability of the membrane [44].

Underwater oil contact angle (UOCA) measurements further demonstrate the oleophobicity in water. The pristine PVDF membrane exhibits strong underwater oleophilicity, where the oil droplet nearly completely wets the surface (UOCA ≈ 0°, Figure 3e). In contrast, the MTSP shows a dramatically increased UOCA of 136° ± 20° (Figure 3f), indicative of pronounced underwater oleophobic behavior. This improvement is primarily attributed to the stable water layer formed by the hydrophilic functional groups on the membrane surface, which effectively reduces direct oil-solid contact and enhances interfacial repulsion against oil droplets. Such behavior is also consistent with previous observations in related fibrous MXene/SiO_2_-modified PVDF membranes, where the combination of hydrophilic functional groups and surface structural effects promoted water-layer formation and suppressed oil adhesion [48]. As a result, oil adhesion is reduced, thereby lowering the risk of membrane fouling during separation.

### 2.3. Oil/Water Separation Performance

The oil/water separation efficiency of the MTSP membrane was evaluated using oil-in-water emulsions under a low transmembrane pressure of 0.1 bar. All flux and separation efficiency data shown in Figure 3g,h were measured under identical experimental conditions, namely vacuum filtration at 0.1 bar and room temperature using surfactant-stabilized oil-in-water emulsions. Figure 3g compares the separation performance of various membranes. The unmodified PVDF membrane is ineffective for emulsified oil removal, achieving only ~57% separation for a kerosene-in-water emulsion. Introducing fibrous MXene into PVDF markedly improves the separation efficiency to ~94%, indicating that MXene incorporation enhances membrane hydrophilicity and facilitates water permeation. The further addition of TP and SiO_2_ increases the oil removal efficiency, and all TP-SiO_2_-MXene composite membranes exhibit >98% separation across the tested composition range. Among them, the membrane with a SiO_2_:TP ratio of 1:1 reaches ~99.5% separation efficiency. However, the differences in oil removal among the tested TP/SiO_2_ ratios are relatively small, whereas the flux varies more noticeably with composition. In particular, increasing TP content tends to reduce the permeation flux. Therefore, rather than being identified solely on the basis of separation efficiency, the 1:1 membrane was selected as the representative composition for further evaluation because it provides a favorable balance between high oil rejection and relatively high flux.

To further clarify the individual and combined contributions of the introduced components, the present results can be compared with our previous fibrous MXene/SiO_2_-modified PVDF system, in which the separation efficiency increased stepwise from ~57% for pristine PVDF to ~85% for PVDF@SiO_2_, ~94% for PVDF@fibrous MXene, and ~99.3% for the fibrous MXene-SiO_2_ co-modified membrane. The corresponding permeate flux of the co-modified membrane reached 2800–3050 L m^−2^·h^−1^. These results indicate that neither inorganic hydrophilic particles nor fibrous MXene alone were sufficient to achieve the best overall performance, whereas co-modification produced a more pronounced enhancement in both separation efficiency and flux. In this context, the further introduction of TP in the present MTSP system is considered to provide additional interfacial functionalization and improved overall separation performance. A comparison of representative multifunctional membranes reported in the literature, including our previous work, is summarized in Table S1.

In addition to high separation efficiency, the MTSP membrane exhibits excellent water permeance. The hydrophilic composite coating significantly increases the pure water flux compared to neat PVDF, reaching on the order of 2200–2900 L·m^−2^·h^−1^ for the various MTSP formulations. Even the optimal 1:1 SiO_2_:TP membrane maintains a high flux (~2420–2670 L·m^−2^·h^−1^) in kerosene emulsion filtration. This flux is one order of magnitude greater than that of the pristine hydrophobic PVDF, highlighting the advantage of the superhydrophilic, macro-porous structure. A trade-off is observed in that the 1:1 membrane shows a moderately lower flux than the SiO_2_:TP = 1:3 membrane, decreasing by approximately 15%, likely due to denser surface coverage and increased transport resistance. Nonetheless, the selected MTSP membrane achieves an excellent balance, with both near-quantitative oil removal and high throughput. The 1:1 composite was selected as the representative membrane for further evaluation because, although the different TP/SiO_2_ ratios showed similarly high separation efficiencies, the flux varied more noticeably with composition. In particular, increasing TP content tended to reduce the permeation flux. Therefore, the 1:1 formulation was considered to provide the best overall balance between oil rejection and permeation performance.

**Figure 4:**
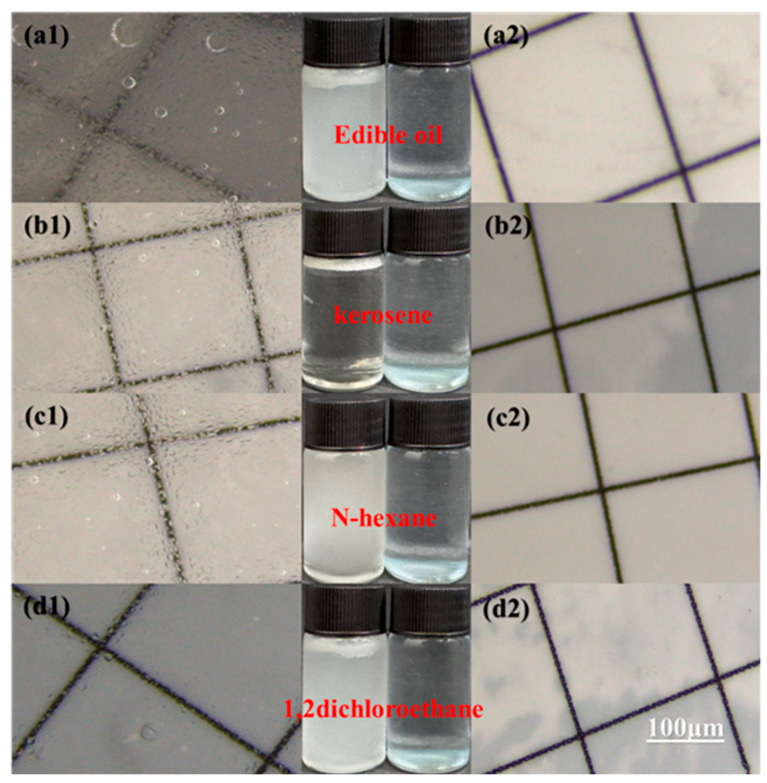
Light microscope images of various oil-in-water emulsions before and after separation. (**a1**–**d1**) represent the feed emulsions before separation, while (**a2**–**d2**) represent the corresponding filtrates after separation.

The stability and versatility of the MTSP membrane were further validated by separating a range of oil-in-water emulsions. As shown in Figure 3h, the optimized membrane consistently achieved separation efficiencies higher than 99% for different oils, including kerosene, edible oil, n-hexane, and 1,2-dichloroethane. In addition, the permeation flux varied with oil type, which was mainly associated with differences in oil viscosity. According to supplier specifications and literature values, n-hexane and 1,2-dichloroethane exhibit relatively low viscosities of approximately 0.31 mPa·s at 20 °C and 0.8 mPa·s at 20 °C, respectively, whereas commercial edible oil typically shows a much higher viscosity of about 49.5 mPa·s at 25 °C. These reference values should be taken as indicative only, since the actual viscosity may vary with temperature, formulation, and batch. Consistent with this trend, the flux for the lower-viscosity oils (kerosene, n-hexane, and 1,2-dichloroethane) was approximately 1700–2045 L·h^−1^ m^−2^, whereas a lower flux of ~1000 L·m^−2^·h^−1^ was obtained for the higher-viscosity edible-oil emulsion. Importantly, even for this more challenging system, the separation efficiency remained above 99%. Optical microscopy images before and after separation further corroborate the demulsification performance. As exemplified by the four oil-in-water emulsions, the feed emulsions appeared milky with abundant micron-sized oil droplets (Figure 4a1–d1, left), while the oil droplets disappeared after filtration and the permeate became transparent (Figure 4a2–d2, right), demonstrating the excellent separation capability of the MTSP membrane. Collectively, these results indicate that the MTSP membrane is applicable to various oily wastewater streams. Its superhydrophilic surface combined with a hierarchical porous architecture enables simultaneously high flux and high rejection across different oils, achieving performance comparable to or exceeding that of recently reported advanced oil/water separation membranes. To place the present results in context, the performance of the MTSP membrane was compared with representative multifunctional oil/water separation membranes reported in the recent literature, as summarized in Table S1. The comparison shows that the MTSP membrane achieves competitive oil/water separation efficiency together with effective phenol and heavy-metal removal, while maintaining relatively high flux under mild operating conditions.

### 2.4. Durability and Stability

**Figure 5:**
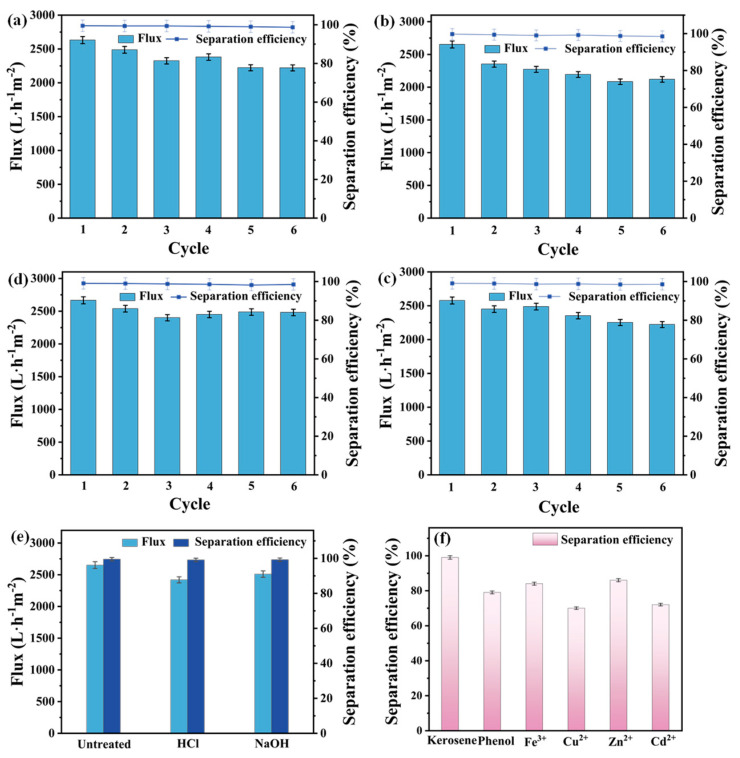
Separation efficiency and flux of (**a**) Kerosene, (**b**) Edible oil, (**c**) N-hexane, (**d**) 1,2-dichloroethane water-in-oil emulsion and (**e**) MTSP membrane under harsh environments. (**f**) Separation efficiency of MTSP membrane in simulating actual oily wastewater treatment.

Practical wastewater treatment demands that membranes maintain performance over repeated use and under harsh conditions. Therefore, the longevity of the MTSP membrane’s separation efficacy was assessed through cyclic reuse tests and chemical stability evaluations. The membrane demonstrated excellent reusability in cyclic oil/water separation. After five consecutive filtration cycles (with cleaning between cycles), the MTSP membrane retained a high separation efficiency in all tested emulsions (kerosene, cooking oil, hexane, dichloroethane). Only a very slight decline (<3% absolute) in oil removal efficiency was observed over five cycles. This minor drop indicates good stability of the superhydrophilic coating and negligible irreversible fouling. The water flux showed a moderate reduction after multiple cycles, but the overall flux decline was small and had a limited impact on separation performance. The flux decay can be attributed to trace amounts of oil droplets that inevitably remain caught in the membrane after each run, gradually accumulating despite cleaning. As expected, the flux loss was more pronounced for the viscous oil (cooking oil) emulsion, since higher viscosity oils tend to leave more residual fouling that is harder to fully remove. In contrast, the lower-viscosity oil emulsions were easier to clean off, resulting in negligible flux reduction. Nonetheless, in all cases, the MTSP membrane continued to exhibit >98–99% separation in cycle 5, confirming that its anti-fouling property effectively preserves performance upon reuse. These cycle tests (Figure 5a–d) demonstrate the membrane’s outstanding operational stability and reusability, which are critical for economic feasibility in industrial separation processes.

Practical application of the MTSP membrane for treating complex oily wastewater requires that it maintain high oil/water separation performance under harsh conditions. To evaluate its chemical stability at extreme pH, the MTSP membrane was immersed in highly acidic (pH = 1) and alkaline (pH = 12) solutions for 24 h, thoroughly rinsed with deionized water, and then re-evaluated for separation performance. As shown in Figure 5e, the pristine MTSP membrane exhibited a water flux of 2420 L·m^−2^·h^−1^ and a separation efficiency of 99.5% for a kerosene-in-water emulsion. After immersion in the pH = 1 solution, the separation efficiency remained as high as 99.1%, with the flux showing only a slight change (2670 L·m^−2^·h^−1^), indicating that the oleophobic function of the membrane was well preserved after exposure to a highly corrosive environment. Similarly, after soaking in a pH = 12 solution, the flux decreased slightly to 2510 L·m^−2^·h^−1^, which may be attributed to minor surface swelling or subtle pore-structure variations, while the separation efficiency was still maintained at 99.2%. The retained performance under both strongly acidic and alkaline conditions highlights the chemical durability of the TP-SiO_2_-fibrous MXene nanoparticles coating. Unlike conventional polymeric or inorganic membranes that are prone to degradation under extreme pH, the hybrid structure in this work remains structurally and functionally stable, likely due to strong hydrogen bonding and physical embedding between TP/MXene and the PVDF matrix, together with the chemical inertness of SiO_2_. Overall, these results demonstrate that the MTSP membrane can sustain high-efficiency separation and high flux under repeated operation and strongly corrosive environments, underscoring its applicability and reliability for long-term treatment of complex wastewater.

### 2.5. Multifunctional Pollutant Removal Performance

Furthermore, the multifunctional pollutant removal capacity of the MTSP membrane is demonstrated in Figure 5, confirming its potential as a comprehensive treatment solution for real wastewater. Beyond oil/water separation, the MTSP membrane demonstrates strong multifunctional capabilities, particularly for the simultaneous removal of heavy metal ions and organic pollutants. This is critical for treating real industrial wastewater that often contains a mixture of oil, metals, and dissolved organics. A simulated complex wastewater system was designed to contain emulsified kerosene, four common heavy metals (Fe^3+^, Cu^2+^, Zn^2+^, Cd^2+^, each at 10 mg·L^−1^), and phenol (20 mg·L^−1^) as a model organic contaminant.

During a single filtration process, the MTSP membrane exhibited excellent overall separation performance. The oil separation efficiency remained high (~99%), while significant adsorption of other pollutants was also achieved. Phenol removal reached ~79%, and heavy metal ion removal efficiencies ranged from 70% to 86%. Zn^2+^ and Fe^3+^ exhibited the highest uptake (~84–86%), while Cu^2+^ and Cd^2+^ showed slightly lower removal (~70–72%) (Figure 5f). These high single-pass efficiencies confirm that the membrane can concurrently filter oil and adsorb multiple dissolved contaminants, positioning it as a promising all-in-one treatment solution.

### 2.6. Mechanism of Simultaneous Adsorption and Separation

The multifunctional performance of the MTSP membrane stems from synergistic regulation of interfacial free energy and chemically active hierarchical architecture within the membrane matrix. Its separation behavior is fundamentally governed by the modulation of the oil-water-solid interfacial energetics. Upon exposure to oil-water emulsions, abundant hydrophilic oxygen-containing groups, phenolic hydroxyls from tea polyphenols, surface -OH terminations on MXene, and silanol groups from SiO_2_ form a thermodynamically favorable hydrogen-bonding network with water molecules [48]. This interaction lowers the solid-water interfacial free energy and drives the spontaneous formation of a stable hydration layer. The hydration layer imposes an energetic barrier against oil penetration by increasing the free-energy cost required to displace interfacial water, thereby repelling oil droplets while maintaining energetically favorable water transport [42]. Its persistence suppresses oil adhesion, mitigates pore blockage, and stabilizes long-term permeation. From a wettability and capillarity standpoint, the observed separation behavior can be understood from the combined effects of surface chemistry and the hierarchical porous structure of the membrane. The superhydrophilic surface minimizes water entry resistance, whereas the high underwater oil contact angle indicates thermodynamically unfavorable oil intrusion [49]. This “water-preferred, oil-rejected” interfacial energy landscape, together with the interconnected porous framework observed in the SEM images, contributes to capillarity-controlled phase selectivity and efficient oil-water separation [50]. Representative macrovoids, interconnected pore channels, and porous substructures are indicated in Figure 1a to facilitate visualization of the membrane morphology.

**Figure 6:**
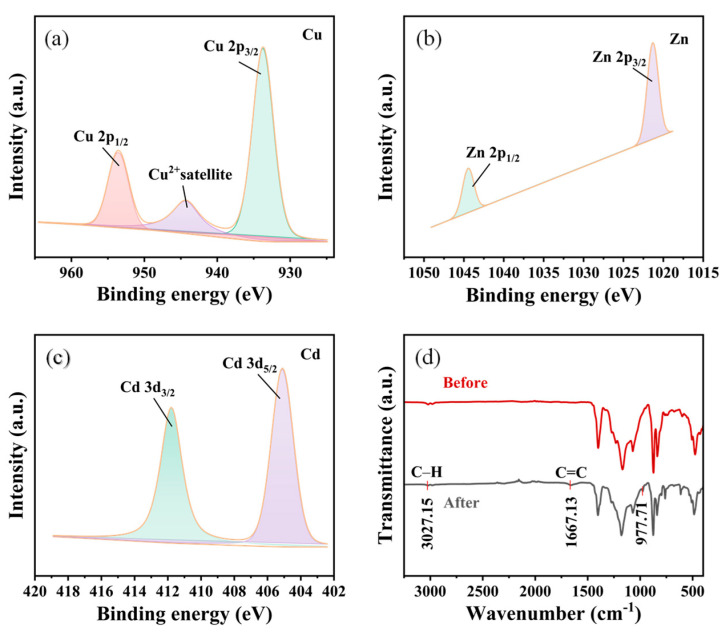
XPS survey spectrum of used MTSP membrane after treatment. (**a**) The Cu 2p peak, (**b**) the Zn 2p peak, and (**c**) the Cd 3d peak of XPS and (**d**) FTIR comparison of the fresh MTSP membrane and the used MTSP membrane after phenol treatment.

**Figure 7:**
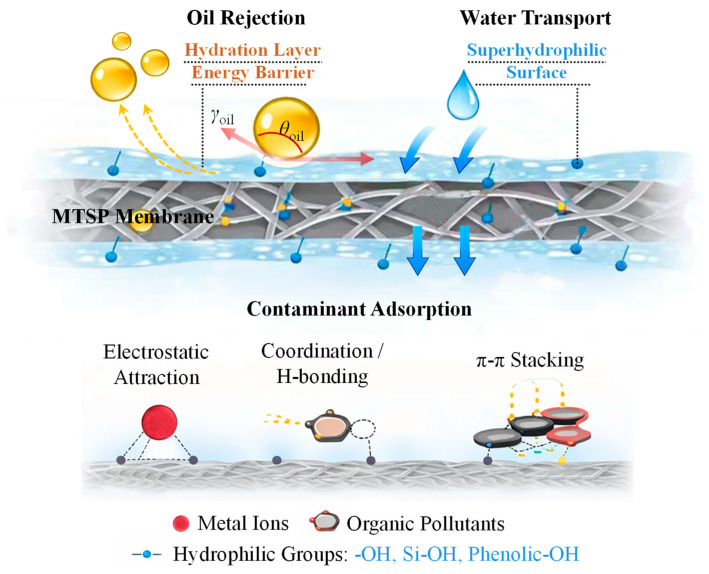
Mechanism of MTSP membrane on oily pollutants and heavy metal ions.

Concurrent removal of dissolved contaminants proceeds via surface-mediated chemical interactions. Negatively charged sites electrostatically attract multivalent metal cations, while phenolic and aromatic moieties enable coordination, hydrogen bonding, and π–π interactions with organic pollutants [51]. The fibrous MXene network further enhances performance by providing high surface area, abundant active terminations, and efficient mass-transfer pathways, thereby accelerating adsorption kinetics. To further verify the proposed adsorption process, post-testing surface characterization was performed on the used membrane after treatment. As shown in Figure 6a–c, characteristic Zn 2p, Cd 3d, and Cu 2p signals were clearly detected on the membrane surface after testing. Specifically, the Zn 2p_3/2_/2p_1/2_ doublet and Cd 3d_5/2_/3d_3/2_ doublet confirm the retention of Zn- and Cd-containing species, while the Cu 2p spectrum, together with the Cu^2+^ satellite feature, indicates that Cu remained on the membrane surface predominantly in the Cu^2+^ state after treatment [52,53,54]. In addition, compared with the fresh membrane, the used membrane after phenol treatment exhibited distinct FTIR changes at around 3027, 1667, and 977 cm^−1^ (Figure 6d). The band at around 3027 cm^−1^ is consistent with aromatic C–H stretching, while the changes near 1667 and 977 cm^−1^ can be associated with aromatic/phenolic-related vibrations and adsorption-induced changes in the local chemical environment of surface functional groups. Taken together, these spectral changes are consistent with the retention of phenolic species on the membrane surface after treatment [55]. Taken together, these spectral changes are consistent with the retention of phenolic aromatic species on the membrane surface after treatment. These post-testing results provide direct evidence that the removal of dissolved pollutants involves not only interfacial attraction in solution, but also effective surface adsorption on the MTSP membrane. These mechanistic interpretations are supported by experimental evidence [56]. The near-zero water contact angle and high underwater oil contact angle confirm favorable water spreading and strong oil repellency, while the high flux under low pressure indicates reduced interfacial transport resistance (Figure 7). The substantial removal efficiencies for phenolic compounds and multivalent metal ions further validate the proposed electrostatic and coordination-driven adsorption mechanisms [57].

## 3. Materials and Methods

### 3.1. Materials

Polyvinylidene fluoride (PVDF, Mw ≈ 900,000) was purchased from Suzhou Punard Plasticization Co., Ltd., Suzhou, China, Tea polyphenols (TP), extracted from green tea and rich in catechins and tannins, were supplied by Shanghai Aladdin Biochemical Technology Co., Ltd., Shanghai, China, and served as a natural hydrophilic modifier owing to their abundant phenolic hydroxyl groups and strong metal chelation ability. SiO_2_ nanoparticles were sourced from Bisley New Materials Limited to enhance surface hydrophilicity and roughness. MXene nanosheets (Ti_3_C_2_T_x_) were obtained from Jiaxing Hesmo New Material Technology Co., Ltd., Jiaxing, China, and prepared by selectively etching the Ti_3_AlC_2_ MAX phase with hydrofluoric acid. All other chemical reagents, including N-methyl-2-pyrrolidone (NMP), ethanol, and deionized water, were purchased from Sinopharm Chemical Reagent Co., Ltd., Shanghai, China.

### 3.2. Preparation of MTSP Membrane

**Figure 8:**
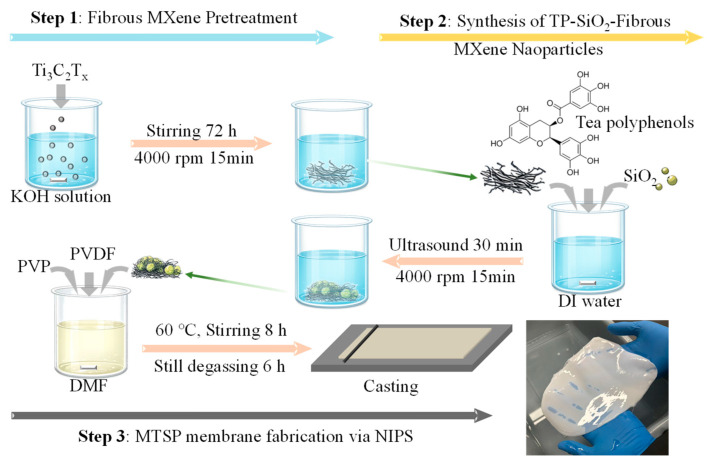
Preparation diagram of MTSP membrane.

The MTSP membrane was prepared through three consecutive steps, including fibrous MXene pretreatment, synthesis of TP-SiO_2_-fibrous MXene composite nanoparticles, and membrane fabrication via nonsolvent-induced phase separation [48]. Briefly, MXene (Ti_3_C_2_T_x_) nanoparticles (0.1 g) were dispersed in 50 mL KOH solution prepared by dissolving 10 g KOH in 50 mL water, and magnetically stirred at room temperature for 72 h. The suspension was then centrifuged at 4000 rpm for 15 min, and the collected precipitate was washed with deionized water and re-centrifuged five times until the supernatant approached neutrality. The resulting solid was dried to obtain fibrous MXene powder. Subsequently, fibrous MXene, TP, and SiO_2_ were dispersed in deionized water and ultrasonicated for 30 min to form a uniform suspension, followed by centrifugation at 4000 rpm and repeated washing with fresh deionized water; the collected particles were then dried to yield TP-SiO_2_-fibrous MXene composite nanoparticles. Unless otherwise specified, the SiO_2_:TP ratio was defined on a mass basis, and for the representative 1:1 formulation, 0.1 g fibrous MXene, 0.1 g TP, and 0.1 g SiO_2_ were used. For other SiO_2_:TP compositions investigated in this work, including 3:1, 2:1, 1:1, 1:2, and 1:3, the masses of TP and SiO_2_ were adjusted accordingly, while the fibrous MXene content was kept constant. To prepare the membrane, the resulting composite nanoparticles were dispersed in 40 mL DMF and ultrasonicated for 90 min. PVDF (3 g) and PVP (0.1 g) were then added, and the mixture was mechanically stirred at 60 °C for 8 h to form a homogeneous casting solution. For the 1:1 formulation, the total filler loading in the casting solution was 0.3 g, corresponding to 10 wt% relative to PVDF. After degassing for 6 h, the solution was cast onto a clean glass plate using a 100 μm casting knife and immediately immersed in a deionized water coagulation bath. The membrane was formed by phase inversion and subsequently stored in deionized water prior to use to prevent drying and maintain structural integrity (Figure 8). For comparison, a series of reference membranes was also prepared under identical conditions, including pristine PVDF and PVDF composites containing only TP, SiO_2_, or MXene. For cyclic separation tests, after each filtration cycle, the membrane was rinsed with ethanol and deionized water to remove residual oil and contaminants before reuse.

### 3.3. Characterization

The membrane’s cross-section and surface morphology were observed by scanning electron microscopy (SEM) (FEI, USA) after sputter-coating with gold. SEM images revealed the distribution of SiO_2_ particles and MXene within the PVDF matrix, as well as the surface texture. Energy-dispersive X-ray spectroscopy (EDS) mapping was performed in SEM to confirm the presence of elemental Ti (from MXene), Si (from SiO_2_), and their dispersion. X-ray diffraction (XRD) was used to identify crystalline phases: PVDF characteristic peaks and any MXene or SiO_2_ peaks, indicating successful incorporation. Fourier-transform infrared spectroscopy (FTIR) was employed to detect chemical bonds; peaks corresponding to polyphenol functional groups (O–H, aromatic C=C), Si–O–Si, and PVDF were analyzed to confirm the chemical interactions in the composite. X-ray photoelectron spectroscopy (XPS) further verified surface chemistry and the presence of elements (C, O, Si, Ti, F), providing evidence of TP and MXene on the membrane surface.

### 3.4. Separation Performance Evaluation

The separation performance of membranes was systematically evaluated using emulsified oil-water mixtures in a filtration configuration. The filtration experiments were conducted using a vacuum filtration setup equipped with an effective membrane filtration area of 19.6 cm^2^. A mild and constant transmembrane pressure of approximately 0.1 bar was applied using a vacuum pump to ensure stable and reproducible conditions during filtration. To simulate realistic industrial wastewater, stable oil-in-water emulsions were prepared by dispersing 0.5 vol% of different oils into deionized water, with the addition of 0.1 wt% Tween-80 as a surfactant. The mixture was subsequently sonicated for 30 min to yield a stable and homogenous milky emulsion. Four representative types of oils were selected to evaluate membrane performance comprehensively, including kerosene (a light hydrocarbon oil, low viscosity), edible vegetable oil (soybean oil, higher viscosity), n-hexane (low viscosity, volatile non-polar solvent), and 1,2-dichloroethane (a dense chlorinated organic solvent), considering their distinct physicochemical properties.

Prior to the tests, the membranes were thoroughly pre-wetted using deionized water. Subsequently, 200 mL of each emulsion was filtered under constant applied pressure. During filtration, the permeate volume and filtration time were recorded, and the permeation flux *J* (L·m^−2^·h^−1^) was calculated according to the equation [48]:

(1) $$J=\frac{V}{A\cdot t}$$

where *V* represents the permeate volume (L), *A* is the effective membrane area (m^2^), and *t* denotes filtration duration (h).

To quantitatively assess oil rejection (separation efficiency), two analytical approaches were employed: UV-Vis spectroscopy and turbidity measurement. For UV-Vis analysis, oil concentration in permeates was determined using solvent extraction with n-hexane, followed by measurement of UV absorbance at an appropriate wavelength. Oil concentration was then calculated based on a standard calibration curve constructed prior to experiments. Oil rejection efficiency (Re) was thus determined as follows [48]:

(2) $$R(\%)=(1-\frac{C_{\mathrm{perm}}}{C_{\mathrm{feed}}})\times 100\%$$

where *C_perm_* and *C_feed_* represent oil concentrations in the permeate and feed emulsion, respectively. Additionally, turbidity analysis was conducted using a turbidimeter to visually confirm the clarity of permeates; successful separation typically yields a permeate turbidity value below 1 NTU. For clear comparison and to highlight the superior performance of the developed MTSP composite membrane, several control membranes, including pristine PVDF, PVDF modified solely with TP, and PVDF modified solely with MXene, were also evaluated under identical experimental conditions. Each filtration experiment was replicated at least three times to ensure data reliability and reproducibility, with mean values and standard deviations reported.

## 4. Conclusions

An organic-inorganic co-modified PVDF membrane was successfully fabricated via nonsolvent-induced phase separation by integrating TP, SiO_2_, and fibrous MXene into the membrane matrix. The synergistic incorporation of these components effectively regulated the surface chemistry and pore structure of PVDF, producing a superhydrophilic underwater-oleophobic interface that is favorable for rapid water transport, efficient oil rejection, and fouling mitigation. The optimized MTSP membrane delivered high oil/water separation efficiency (~99.5%) and high flux (2420–2670 L·h^−1^ m^−2^) at 0.1 bar, while maintaining stable performance during repeated operation and under strongly acidic and alkaline conditions. Notably, for complex simulated wastewater containing emulsified oil, phenol, and multiple heavy metal ions, the membrane preserved ~99% oil/water separation efficiency and enabled the simultaneous removal of dissolved organic and inorganic pollutants, demonstrating clear potential for one-step treatment of composite wastewater. Overall, this work provides a simple and scalable co-modification strategy for constructing PVDF-based membranes that integrate high flux, antifouling behavior, and multipollutant removal, offering a promising route for the advanced treatment of complex oily wastewater.

## Data Availability

All relevant data are included in the paper.

## Supplementary Materials

The following supporting information can be downloaded at: https://www.mdpi.com/article/10.3390/molecules31081372/s1, Figure S1: (**a**) Enlarging spectrum of the 3000–3700 cm^−1^ region. (**b**) Enlarging spectrum of the 900–1200 cm^−1^ region; Table S1: Comparison of different multifunctional membranes. References [58,59,60,61,62,63,64,65,66] are cited in the supplementary materials.
