# Supplementary material for: Organic-Inorganic Co-Modified PVDF Membrane for High-Flux Oil/Water Separation and Simultaneous Multi-Pollutant Removal
Source: Molecules. 2026 Apr 21;31(8):1372. doi: 10.3390/molecules31081372 (PMC13119194; doi:10.3390/molecules31081372)
Supplement: Supplementary file 1 [file molecules-31-01372-s001.zip › molecules-4216203-supplementary.pdf]

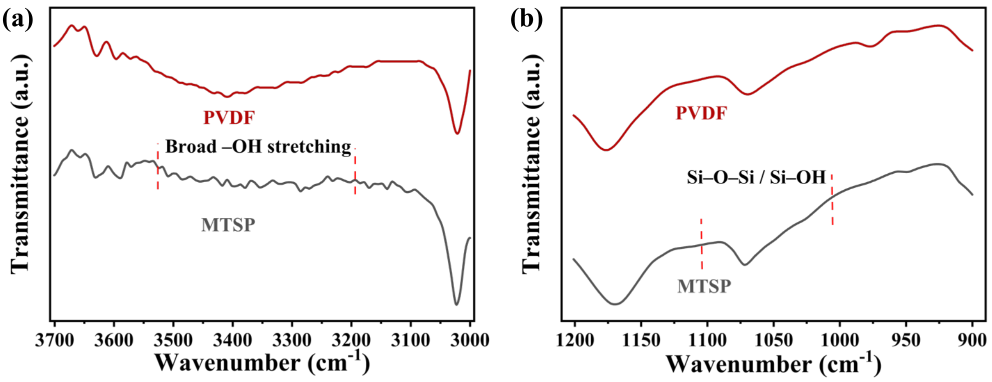

Figure S1. (a) Enlarging spectrum of the 3000-3700 cm^{-1} region. (b) Enlarging spectrum of the 900-1200 cm^{-1} region.

Table S1. Comparison of different multifunctional membranes

Material	Separation efficiency (%)	Flux ($L \cdot m^{-2} \cdot h^{-1}$)	Heavy metal removal	Organic pollutant / dye removal	Reference
**MTSP membrane**	>99	1700-2045 (low-viscosity oils); ~1000 (edible oil emulsion)	Fe^{3+}: ~78%; Cu^{2+}: ~60%; Zn^{2+}: ~88%; Cd^{2+}: ~70%	Phenol: ~79%	This work
**MMP membrane**	>99.3	2800-3050	Fe^{3+}: 78%; Cu^{2+}: 60%; Zn^{2+}: 88%; Cd^{2+}: 70%; Pb^{2+}: 85%	MB: 98.7%; MG: 95%	[1]
**TA-APTES-SA-PVDF**	<90.0	6364	Cu^{2+}: <70.0%		[2]
**PPs-coated PVDF membrane**	>99	7994 ± 150		MB: 99.75%	[3]
**PANI@CA**	>99	125-230	Cu^{2+}: 57.5%; Cd^{2+}: 71.2%; Pb^{2+}: 98.8%	CV: 96.9%; MB: 99.4%; CR: 83.2%; MO: 86.2%	[4]
**pDA/PEI-G-SA**	>99	235.67-1266.95	Cu^{2+}: 64.3%	MB: 92.9%; MG: 94.6%; CV: 97.3%	[5]
**PCN-224/TA/PVDF**	>99	829-1542		MB: 98.5%; RhB: 99.4%	[6]
**SiO_2/GO**	>99	470		MB: >98.5 %	[7]

Material	Separation efficiency (%)	Flux ($L\cdot m^{-2}\cdot h^{-1}$)	Heavy metal removal	Organic pollutant / dye remova	Reference
**PSBMA/HNTs/PVDF membrane**	>99.7	201.3		Methyl blue: 96.8%; Orange G: 92.7%	[8]
**Modified-MOF-808-loaded PAN membrane**	>99.97	329.3	Cu^{2+}: 83.1–96.5%; Cd^{2+}: 80.4–92.6%; highest heavy-metal removal: 97.7%		[9]
**QCD-MMT-TA coated PVDF membrane**	>94.2	5017.9	Cu^{2+}: 63.2%		[10]

References

1. Xu, X.; Cheng, S.; Lu, Z.; Li, P.; Xue, Y.; Yang, Y.; Ni, T.; Teng, J. Bioinspired multi-functional modified PVDF membrane for efficient oil-water separation. *Sep. Purif. Technol.* **2025**, 358, 130410.

2 Yan, L.L.; Yang, X.B.; Zeng, H.Z.; Zhao, Y.Y.; Li, Y.X.; He, X.Z.; Ma, J.; Shao, L. Nanocomposite hydrogel engineered hierarchical membranes for efficient oil/water separation and heavy metal removal. *J. Membr. Sci.* **2023**, *668*, 121243.

3 Wu, M.M.; Mu, P.; Li, B.F.; Wang, Q.T.; Yang, Y.X.; Li, J. Pine powders-coated PVDF multifunctional membrane for highly efficient switchable oil/water emulsions separation and dyes adsorption. *Sep. Purif. Technol.* **2020**, *248*, 117028.

4 Wang, C.Z.; Wang, F.F.; Zhang, H.; Zhang, Y.N.; Zhang, C.G.; Zang, W.; Peng, M.J.; Yang, Y.Y.; Wang, S.W.; Xu, C.; Wu, A.G.; Zhang, Y.J. Multifunctional polyaniline modified calcium alginate aerogel membrane with antibacterial, oil-water separation, dye and heavy metal ions removal properties for complex water purification. *Sci. Total Environ.* **2024**, *927*, 172058.

5 Wang, Z.C.; Guan, M.; Yang, X.; Li, H.Z.; Zhao, Y.; Chen, Y.L. A trifecta membrane modified by multifunctional superhydrophilic coating for oil/water separation and simultaneous adsorption of dyes and heavy metal. *Sep. Purif. Technol.* **2024**, *333*, 125904.

6 Xue, J.J.; Xu, M.J.; Gao, J.M.; Zong, Y.Q.; Wang, M.X.; Ma, S.S. Multifunctional porphyrinic Zr-MOF composite membrane for high-performance oil-in-water separation and organic dye adsorption/photocatalysis. *Colloids Surf. A Physicochem. Eng. Asp.* **2021**, *628*, 127288.

7 Liu, Y.; Zhang, F.R.; Zhu, W.X.; Su, D.; Sang, Z.Y.; Yan, X.; Li, S.; Ji, L.; Dou, S.X. A multifunctional

hierarchical porous SiO_2/GO membrane for high efficiency oil/water separation and dye removal. *Carbon* **2020**, *160*, 88–97.

8 Zhang, G.; Li, Y.; Gao, A.; Zhang, Q.; Cui, J.; Zhao, S.; Zhan, X.; Yan, Y. Bio-inspired underwater superoleophobic PVDF membranes for highly-efficient simultaneous removal of insoluble emulsified oils and soluble anionic dyes. Chem. Eng. J. **2019**, 369, 576–587.

9 Chen, X.; Chen, D.; Li, N.; Xu, Q.; Li, H.; He, J.; Lu, J. Modified-MOF-808-loaded polyacrylonitrile membrane for highly efficient, simultaneous emulsion separation and heavy metal ion removal. *ACS Appl. Mater. Interfaces* **2020**, *12*, 39227–39235.

10 Xia, D.; Pan, H.; Yang, C.; Chen, Z.; Liao, M.; Lin, Q. One-step separation of oil-water emulsion with heavy metal ions by PVDF membrane modified with zwitterionic coating. *Colloids Surf. A Physicochem. Eng. Asp*. **2024**, 701, 134855.
